# Infectious diseases among elderly persons: Results from a population-based observational study in Shandong province, China, 2013-2017

**DOI:** 10.7189/jogh.11.08010

**Published:** 2021-12-25

**Authors:** Wan-Yu Du, Chao-Nan Yin, Hai-Tao Wang, Zhen-Wei Li, Wen-Jing Wang, Fu-Zhong Xue, Lin Zhao, Wu-Chun Cao, Cheeloo EcoHealth Consortium (CLEC)

**Affiliations:** 1 Institute of EcoHealth, School of Public Health, Cheeloo College of Medicine, Shandong University, Jinan, China; 2 Department of Biostatistics, School of Public Health, Cheeloo College of Medicine, Shandong University, Jinan, China; 3 Department of Occupational Health and Occupational Medicine, School of Public Health, Cheeloo College of Medicine, Shandong University, Jinan, China; 4 State Key Laboratory of Pathogen and Biosecurity, Beijing Institute of Microbiology and Epidemiology, Beijing, China

## Abstract

**Background:**

The health of the elderly is one of the major challenges in today’s ageing society. However, research on infectious diseases among the elderly is limited. This study aimed to describe the epidemiological characteristics and dynamics of infectious diseases among the elderly population aged ≥60 years in Shandong province, China.

**Methods:**

Incidence data for infectious diseases were collected from the Shandong Multi-Center Healthcare Big Data Platform from January 2013 to June 2017, which involved 550 432 elderly persons. We compared the incidence of each infectious disease and disease category, stratified by age, gender, and region. Annual percentage change (APC) was estimated using logarithmic linear regression to examine the incidence trends. Poisson regression was conducted to identify the effect of demographic factors on incidence, with incidence rate ratio (IRR) and their 95% confidence intervals (CIs) estimated.

**Results:**

A total of 27 595 cases of 102 infectious diseases were reported during the study period, with an overall incidence of 1425.51/100 000 person-years. The most common infectious diseases were respiratory and mucocutaneous diseases among the elderly persons, with annual increases of 17.45% and 20.44%, respectively (both *P*<0.05). In rural areas, the incidence of respiratory, gastrointestinal, blood- and sex-transmitted, and mucocutaneous infections increased significantly, with APCs of 178.52%, 204.66%, 28.24%, 63.01%, respectively (all *P*<0.05). Elderly males had a higher risk of infections than that of females, with the highest IRRa of 2.94 (95% confidence interval (CI) = 2.89, 3.00) in respiratory diseases. The elderly aged 85-89 years had a much higher risk of respiratory diseases than those aged 60-64 years (IRRa = 9.85, 95%CI: 9.39, 10.33); however, the risk of blood- and sex-transmitted diseases was highest among the elderly aged 65-69 years (IRRa = 1.24, 95% CI = 1.06, 1.45).

**Conclusions:**

Ageing population are facing a substantial challenge on infectious diseases. More attention should be paid to infections with significant growth. Targeted strategies and measures on elderly persons in different regions and subgroups are urgently needed.

With the increasing longevity and declining fertility rates, the world is ageing in the new century [1]. China is the most populous country worldwide, and had officially entered into an ageing society since 1999. By the end of 2017, the elderly population aged 60 years and above has reached 240 million in China, accounting for 17.3% of the total population [2]. The health of the elderly is threatened by both chronic diseases and infectious diseases due to immune dysfunction, malnutrition, and physiological changes, and infections in the elderly are more frequent and severe [3,4].

The older adults are at significant higher risk for morbidity and mortality due to various infectious diseases. According to the China Health Statistics Yearbook, a total of 4 205 343 patients with pneumonia discharged from public hospitals in 2018, of them 18.0% were aged over 60 years [5]. From 1990 to 2018, the influenza-related excess mortality rates ranged from 30.8 to 170.2 per 100 000 people among the elderly in China [6]. In addition, it has been estimated that the global incidence of herpes zoster in the general population is 3/1000 to 5/1000 person-years, with the incidence and severity increasing sharply after 50 years of age [7]. These figures highlight the challenges and need for prevention and control of infectious diseases among the elderly population. While previous studies have mainly focused on chronic diseases in the elderly; less is known about their epidemiological characteristics of infectious diseases. Furthermore, current understanding on infectious diseases among the elderly in China is based on the National Notifiable Infectious Disease Surveillance System. Related data on non-notifiable infectious diseases, such as herpes zoster and pneumonia, are still limited [8,9].

As the rapid emergence of big data and data science research, the availability of electronic records has been increased. At present, some health data platforms have been established worldwide [10,11]. Massive data from multiple health-related sources provide us an opportunity to explore the infectious diseases among the elderly persons. In this study, we used dataset extracted from Shandong Multi-Center Healthcare Big Data Platform (SMCHBDP) to describe the epidemiological characteristics and changes of infectious diseases among the elderly in Shandong province, China, identify the most common diseases in different regions and subgroups of populations so as to improve the prevention and control practices of infectious diseases among the elderly.

## METHODS

### Establishment and design of big data platform

“Shandong Multi-Center Healthcare Big Data Platform” (SMCHBDP) [12] is a hybrid system developed by the Health Commission of Shandong Province in 2017. Multi-stage sampling was adopted to obtain the potentially eligible research participants in rural and urban areas of Shandong province according to the proportion of population capacity, whose resident identity card numbers were imported in the big data platform. Multiple sources of health-related data including electronic medical health records, basic public health records, and resident medical insurance payment systems were linked by the unique identity card number. After the data cleaning (removing duplicate or incorrect records), a total of 3 987 573 eligible participants were included (Figure S1, Table S1 in the **Online Supplementary Document**). The platform followed up since the enrolment of participants (finishing baseline filling date).

### Data collection

The population in this study was derived from the platform. All information involving demographic characteristics (sex, age, and residence location) and medical history were integrated by the unique identity card number. The outcome of interest was the occurrence of specific infectious diseases. We used following criteria to further select the elderly subjects: registration into the SMCHBDP between 1 January 2013 and 30 June 2017; age of 60 or more when enrolment; and having sufficient baseline information.

Clinical diagnosis and classification of notifiable infectious diseases were defined by the Law of the People’s Republic of China on the Prevention and Treatment of Infectious Diseases, and other infections were defined according to the International Classification of Diseases-10 (ICD-10). All infectious diseases were classified as respiratory, gastrointestinal, vector-borne, blood- and sex-transmitted, and mucocutaneous diseases based on their main transmission route. This study was approved by the Ethics Committee of the School of Public Health, Shandong University. Researchers can only use the encrypted data on the SMCHBDP server after approval by the official review committee.

### Statistical analysis

Descriptive analyses were used to present the incidences and changes of all infectious diseases among the elderly from 2013 to 2017. Skewed data were expressed as median (interquartile range, IQR). Categorical variables were reported as frequency (n) and proportion (%). The observation period, from the date of enrolment (finishing baseline filling) to the exact date of diagnosis, death, or the end of the study, was computed as the time passed. Person-years (PY) were calculated by adding up the observation period of each individual, based on personal information (enrolment date, diagnosis date, or death date). The incidence density was calculated as the number of new cases that occurred during the study period over the total PY of observation, and expressed as cases per 100 000 PY. We calculated the incidence density of each disease and disease category, stratified by sex, age, and area (urban or rural; Jiaodong Peninsula, central Shandong, northern Shandong or southern Shandong). Seven groups were divided according to the age at diagnosis: 60-64, 65-69, 70-74, 75-79, 80-84, 85-89, ≥90 years. Comparisons of incidence density between different age and gender groups were tested using Miettinen’s formula [13].

Logarithmic linear regression [14] was performed to examine the incidence trends of each disease from 2013 to 2017. Due to the large difference in numerical scale of infectious disease incidence in this study, a required logarithmic transformation was used to approximate a normal distribution (*Y*=ln(r), r is the incidence density), and the linear regression was described as:

*Y = α + βx + ε*

α is the constant term; β is the regression coefficient; ε is the random error.

Y is the natural log of the incidence density in year x and the annual percentage change (APC) can be inferred from the regression coefficient β as: APC = 100 × (e^β^ – 1).

The effect of demographic characteristics on incidence density was tested by *Poisson* regression analysis [15]. Gender, age group of diagnosis, and residence were regarding as influence factor and included into analysis. Univariate *Poisson* regression analysis was used to identify potential factors associated with the incidence of infectious diseases. To control potential confounders, those factors were all included in the multivariate regression model. Incidence rate ratio (IRR) and 95% confidence intervals (CI) were used to assess the risk of developing infections.

Thematic maps were visualized using ArcGIS 10.3 (ESRI, Redlands, CA, USA). Data management and analysis were performed using R software (version 3.4.1, R Foundation for Statistical Computing, Vienna, Austria). A two-sided *P*-value less than 0.05 was considered statistically significant.

## RESULTS

### General information and infections of all subjects

From January 2013 to June 2017, a total of 550 432 elderly aged ≥ 60 years were enrolled in this study. Detailed information on characteristics of all subjects is shown in **Table 1**. Among the elderly, 46.16% were males and 56.23% were living in urban regions. The median age at enrolment was 67 (IQR = 63, 73) years.

**Table 1:** General characteristics and infections of study participants

	Total (N=550 432)	Male (n=254 083)	Female (n=296 349)
**Age at enrolment (years):**			
Median (IQR)	67 (63-73)	66 (62-73)	67 (63-74)
**Age composition at enrolment (years) n (%):**			
60-	344 836 (62.65)	162 848 (64.09)	181 988 (61.41)
70-	151 020 (27.44)	69 911 (27.52)	81 109 (27.37)
80-	49 528 (9.00)	19 746 (7.77)	29 782 (10.05)
90-	5048 (0.92)	1578 (0.62)	3470 (1.17)
**Residence n (%):**			
Urban region	309 511 (56.23)	141 337 (55.63)	168 174 (56.75)
Rural region	240 921 (43.77)	112 746 (44.37)	128 175 (43.25)
**Annual incidence density of infections (per 100** **000 PY):**			
2013	1106.35	1418.61	838.34
2014	922.61	1239.10	651.82
2015	1468.78	1802.73	1184.32
2016	1713.58	2060.99	1419.02
2017	1926.93	2321.82	1592.05
Overall	1425.51	1763.33	1137.66
**Incidence density of infectious diseases by reporting type (per 100 000 PY):**			
Notifiable infectious diseases	978.20	1286.46	715.55
Non-notifiable infectious diseases	447.30	476.87	422.11
**Incidence density of infectious disease by transmission route (per 100 000 PY):**			
Respiratory	923.44	1214.59	675.36
Gastrointestinal	131.52	146.53	118.73
Vector-borne	8.78	11.00	6.89
Blood- and sex-transmitted	71.13	91.51	53.77
Mucocutaneous	290.63	299.69	282.91

IQR – interquartile range, PY – person-years

During the study period, 27 595 cases of 102 infectious diseases were reported in the elderly persons. The overall annual incidence density was 1425.51/100 000 PY, with 1763.33/100 000 PY in males and 1137.66/100 000 PY in females (*P* < 0.001). The annual incidence density of notifiable infectious diseases was 978.20/100 000 PY, and males had a significantly higher incidence density (1286.46/100 000 PY) than females (715.55/100 000 PY) (*P* < 0.0001). For non-notifiable infectious diseases, the overall incidence density was 447.30/100 000 PY, and there were no significant differences between two genders (*P* = 0.07). Among the five categories, the most common infections were respiratory diseases, with the incidence density of 923.44/100 000 PY, followed by mucocutaneous diseases (290.63/100 000 PY), gastrointestinal diseases (131.52/100 000 PY), blood- and sex-transmitted diseases (71.13/100 000 PY), and vector-borne diseases (8.78/100 000 PY) (**Table 1**).

### Temporal distribution and trends of infectious diseases

**Figure 1:**
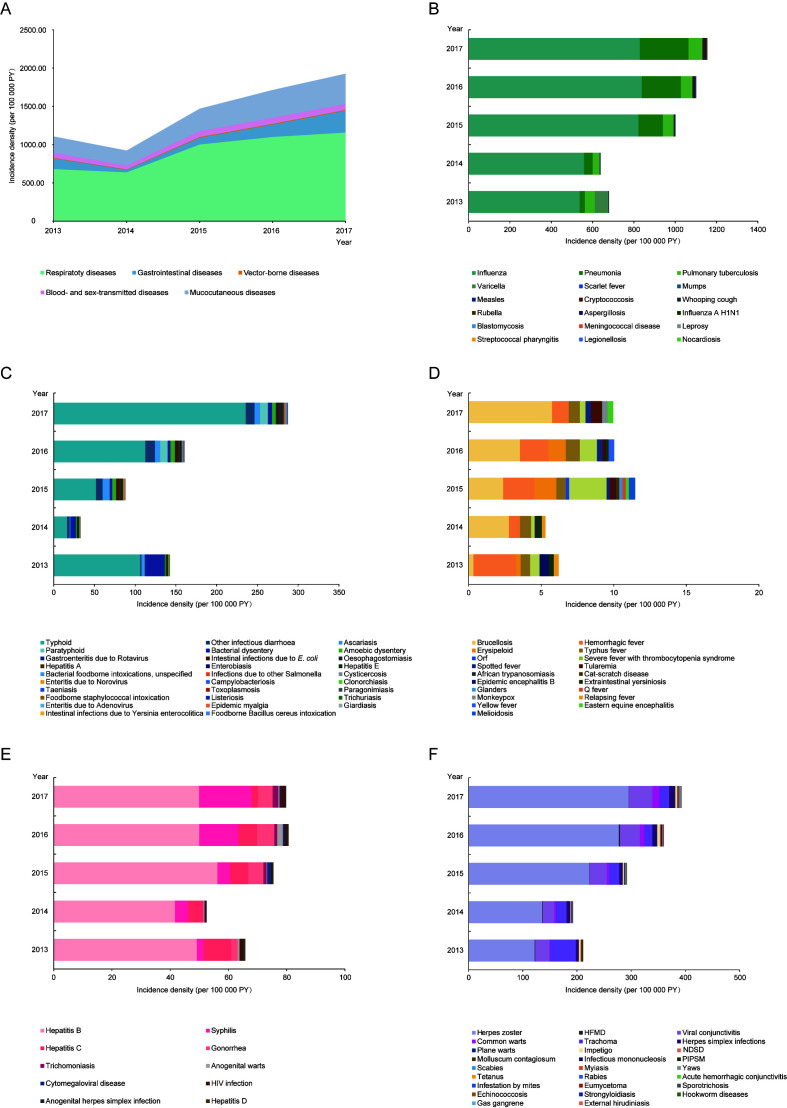
Trends in incidence density and proportions of 102 infectious diseases by categories, 2013-2017. (**A**) Five categories of infectious diseases. (**B**) Respiratory infectious diseases. (**C**) Gastrointestinal infectious diseases. (**D**) Vector-borne infectious diseases. (**E**) Blood- and sex-transmitted infectious diseases. (**F**) Mucocutaneous infectious diseases. HFMD – hand, foot, and mouth disease, NDSD – non-dermatophyte superficial dermatomycoses, PIPSM – picornavirus infections presenting in the skin or mucous membranes.

**Figure 1** and Table S2 in the **Online Supplementary Document** illustrated the secular trends in incidence density of 102 infectious diseases by five categories from 2013 to 2017. The incidence of each disease category increased during the study period, even though a slightly decline occurred between 2013 and 2014 (**Figure 1**, Panel A). Specifically, the incidence density of respiratory and mucocutaneous diseases increased noticeably, with APCs of 17.45% (*P* = 0.03) and 20.44% (*P* = 0.02), respectively (Table S2 in the **Online Supplementary Document**). However, stable trends for gastrointestinal, vector-borne, and blood- and sex-transmitted diseases were observed from 2013 to 2017 (*P* > 0.05).

As shown in **Figure 1**, panel B, influenza was the most common respiratory disease among the elderly population, and maintained a high endemic level. The incidence of pneumonia increased sharply from 25.39/100 000 PY in 2013 to 235.79/100 000 PY in 2017 (APC = 81.55%, *P* = 0.005) (**Figure 1**, Panel B and Table S2 in the **Online Supplementary Document**). Additionally, the incidence of cryptococcosis, whooping cough, and aspergillosis represented significant increased trends (all *P*<0.05) (Table S2 in the **Online Supplementary Document**). The most frequent gastrointestinal infection in the elderly was typhoid. Although the incidence density of typhoid showed an obvious increase from 2014 to 2017, the APC of 42.22% was statistically insignificant (**Figure 1**, Panel C and Table S2 in the **Online Supplementary Document**). Among all blood- and sex-transmitted diseases, hepatitis B remained the most common infection, but syphilis demonstrated the largest increase from 2.28/100 000PY in 2013 to 18.02/100 000PY in 2017 (APC = 68.45%, *P* = 0.01) (**Figure 1**, Panel E and Table S2 in the **Online Supplementary Document**). The incidence of herpes zoster remained an upward trend, with the APC of 28.16% (*P* = 0.008) (**Figure 1**, Panel F and Table S2 in the **Online Supplementary Document**). Among all infections, influenza was the leading disease in each year, followed by herpes zoster. Since 2014, pneumonia has consistently ranked as the third prevalent disease. Additionally, syphilis rose rapidly from 18^th^ in 2013 to 8^th^ in 2017 among the elderly (Table S3 in the **Online Supplementary Document**).

### Geographical distribution and changes of infectious diseases

**Figure 2:**
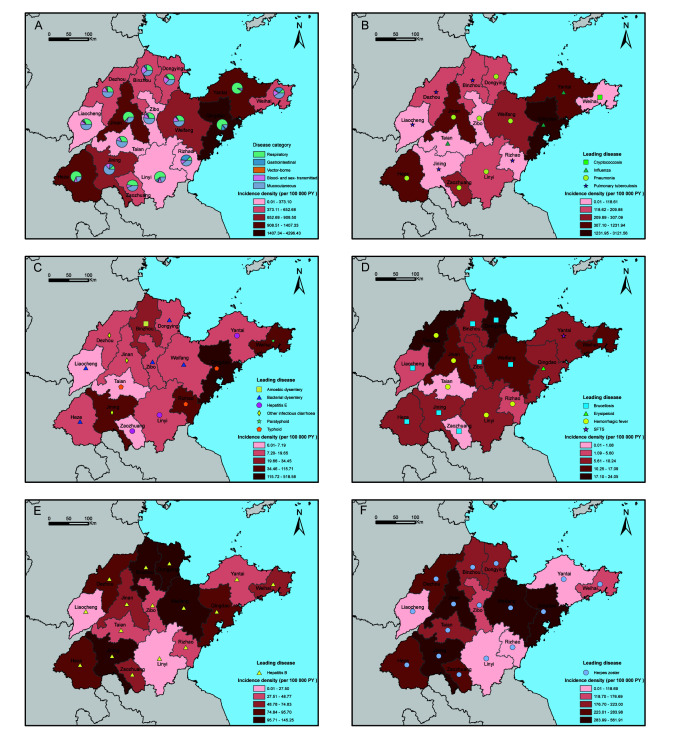
Geographical distribution of infectious diseases by categories in Shandong province, China. (**A**) Proportions of incidence density for disease categories in each prefecture-level city of Shandong province, China. (**B-F**) Geographical distribution of respiratory, gastrointestinal, vector-borne, blood- and sex-transmitted, and mucocutaneous diseases. The symbols in the thematic maps indicate the leading infectious disease of this category in each prefecture-level city. Leading diseases is defined as the disease with the highest incidence density. SFTS – severe fever with thrombocytopenia syndrome

The overall incidence of infectious diseases varied greatly between different prefecture-level cities of Shandong province, China. In **Figure 2**, Panel A, the top three cities with the highest overall incidence were Qingdao (4296.43/100 000 PY), Jinan (1407.33/100 000 PY), and Yantai (1386.56/100 000 PY). In most cities, respiratory and mucocutaneous infections together accounted for more than three quarters of all cases.

For respiratory diseases, the top three cities with the highest incidence were Qingdao (3121.56/100 000 PY), Yantai (1231.94/100 000 PY), and Jinan (881.94/100 000 PY) (**Figure 2**, Panel B). The highest incidence of influenza was mainly located in the cities of Jiaodong Peninsula, accounting for more than 95% of all influenza cases. Pneumonia was the most prevalent respiratory disease in central and southern Shandong province. In northern Shandong, tuberculosis was the most common respiratory infection among the elderly.

As shown in **Figure 2**, Panel C, three cities located in the southeast coastal area of Shandong province had the highest incidence of gastrointestinal diseases – Qingdao (518.58/100 000 PY), Weihai (115.71/100 000 PY), and Rizhao (105.18/100 000 PY). The leading diseases in these cities were typhoid and paratyphoid, accounting for 96.78% of the total cases. For inland area of Shandong province, the leading gastrointestinal infections varied between cities, involving bacterial dysentery, other infectious diarrhoea, typhoid, hepatitis E, and amoebic dysentery.

Vector-borne diseases were relatively rare among the elderly in Shandong province (**Figure 2**, panel D). Brucellosis was the dominant disease in the majority of cities, with the highest incidence in Dongying (24.05/100 000 PY). Blood- and sex-transmitted diseases were prevalent in the central and northern regions of Shandong province, such as Weifang (145.25/100 000 PY), Binzhou (141.54/100 000 PY), and Dongying (133.36/100 000 PY) (**Figure 2**, Panel E); and hepatitis B was the most common disease throughout the province. Additionally, Qingdao had the highest incidence density of mucocutaneous diseases (561.91/100 000 PY); and herpes zoster was the leading disease in all cities of Shandong province (**Figure 2**, Panel F).

Urban and rural areas in Shandong province had different incidence trends for the five categories of infectious diseases. The incidence of infectious diseases in rural areas was generally low in 2013-2014 but increased rapidly in 2015-2017, with respiratory diseases, gastrointestinal diseases, and vector-borne diseases catching up with the incidence in urban areas. The incidence of respiratory, gastrointestinal, blood- and sex-transmitted, and mucocutaneous infections in rural areas depicted increasing trends from 2013 to 2017, with APCs of 178.52% (*P* = 0.03), 204.66% (*P* = 0.006), 28.24% (*P* = 0.01), and 63.01% (*P* = 0.03), respectively. In the Jiaodong Peninsula, the annual incidence of the five categories of infections has remained at a relatively high level, with only a significant increase in the incidence of respiratory infections (APC = 17.15%, *P* = 0.02). In central Shandong, the incidence of mucocutaneous diseases has increased dramatically from 129.47/100 000 PY to 453.17/100 000 PY (APC = 41.33%, *P* = 0.007). A noticeable rise occurred in respiratory, blood- and sex-transmitted, and mucocutaneous infections in northern Shandong, with annual average increases of 35.28% (*P* = 0.005), 17.15% (*P* = 0.02), and 23.75% (*P* = 0.005), respectively. In southern Shandong, the annual incidences of respiratory, gastrointestinal, and mucocutaneous infections have increased significantly, with APCs of 83.80% (*P* = 0.007), 76.93% (*P* = 0.02), and 37.09% (*P* = 0.008), respectively (**Table 2**).

**Table 2:** Annual incidence and trends of the five categories of infectious diseases by regions in Shandong province, China, 2013-2017

Disease category	Regions	Incidence density (per 100 000 PY)					APC (%)
**2013**	**2014**	**2015**	**2016**	**2017**
Respiratory	Urban	1204.96	1080.23	1116.02	1039.81	1135.84	-1.55
	Rural	32.70	55.05	854.53	1180.2	1183.57	178.52*
	Jiaodong Peninsula†	1465.92	1510.2	2177.47	2487.6	2520.13	17.15*
	Central Shandong‡	121.95	173.21	707.75	640.88	658.99	59.72
	Northern Shandong§	68.35	70.16	121.83	159.20	205.55	35.28**
	Southern Shandong‖	52.88	54.28	182.87	313.13	461.77	83.80**
Gastrointestinal	Urban	250.54	51.88	104.22	123.10	115.08	-6.69
	Rural	9.45	8.78	68.05	207.88	509.75	204.66**
	Jiaodong Peninsula	319.73	67.27	192.22	393.11	765.12	42.06
	Central Shandong	3.01	7.48	11.15	19.56	12.11	45.43
	Northern Shandong	11.39	16.19	22.15	17.18	26.81	19.38
	Southern Shandong	10.89	13.29	51.51	71.80	81.24	76.93*
Vector-borne	Urban	8.84	3.55	11.54	12.35	6.81	7.52
	Rural	2.91	7.61	11.34	7.11	14.04	36.06
	Jiaodong Peninsula	6.03	3.30	12.61	11.51	7.95	19.75
	Central Shandong	10.54	4.98	12.17	12.44	13.84	15.72
	Northern Shandong	4.56	8.10	18.46	12.60	15.64	33.73
	Southern Shandong	3.11	6.65	4.29	4.44	5.70	8.41
Blood- and sex-transmitted	Urban	96.68	68.73	91.91	96.29	88.52	1.62
	Rural	27.62	31.04	54.24	60.85	68.43	28.24*
	Jiaodong Peninsula	74.65	49.46	87.10	79.43	88.55	8.49
	Central Shandong	58.71	57.32	65.91	76.44	46.70	-1.68
	Northern Shandong	66.07	74.21	76.30	93.91	129.59	17.15*
	Southern Shandong	54.43	35.45	66.11	76.99	64.13	11.67
Mucocutaneous	Urban	345.45	294.45	359.57	434.90	499.14	11.92
	Rural	47.24	59.15	205.62	266.51	256.19	63.01*
	Jiaodong Peninsula	332.55	269.73	379.63	408.08	450.67	10.76
	Central Shandong	129.47	181.94	289.99	472.00	453.17	41.33**
	Northern Shandong	123.02	122.78	168.60	214.17	270.35	23.75**
	Southern Shandong	108.87	131.83	254.99	300.54	349.17	37.09**

APC – annual percentage change, PY – person-years

*P ≤ 0.0

Note: If the APC is significant (*P*≤0.05), the incidence trend is identified as an increase or decrease; otherwise, the incidence is maintained stable

**P < 0.01.

†JiaodongPeninsula: Qingdao, Yantai, and Weihai.

‡Central Shandong: Jinan, Zibo, Weifang, and Taian.

§Northern Shandong: Liaocheng, Dezhou, Binzhou, and Dongying.

‖Southern Shandong: Jining, Heze, Zaozhuang, Linyi, and Rizhao.

### Demographic characteristics of infectious diseases

**Figure 3:**
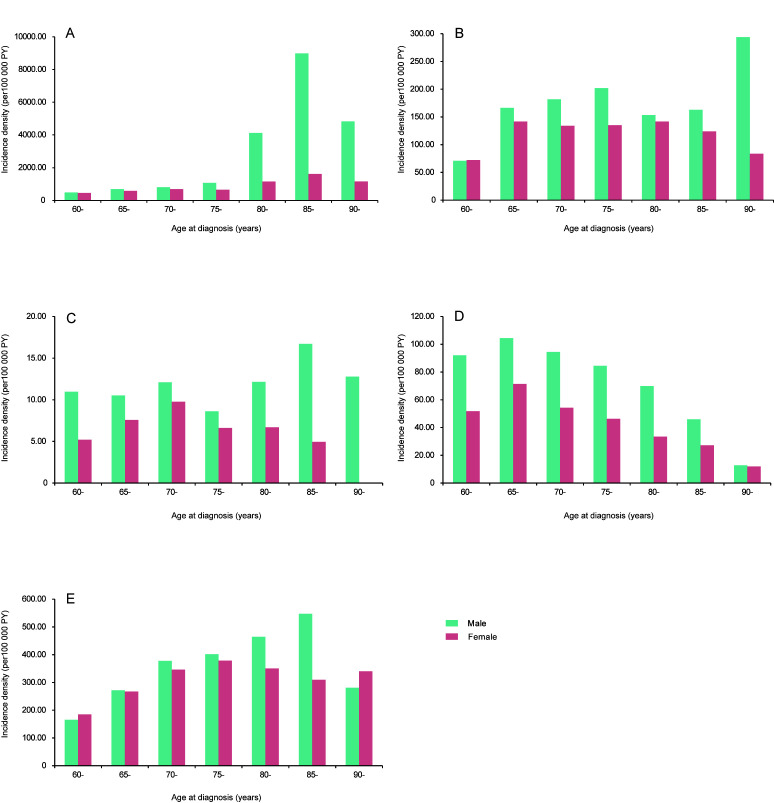
Age and sex differences related to the incidence density of infectious diseases by five categories. (**A**) Respiratory infectious diseases. (**B**) Gastrointestinal infectious diseases. (**C**) Vector-borne infectious diseases. (**D**) Blood- and sex-transmitted infectious diseases. (**E**) Mucocutaneous infectious diseases.

**Figure 4:**
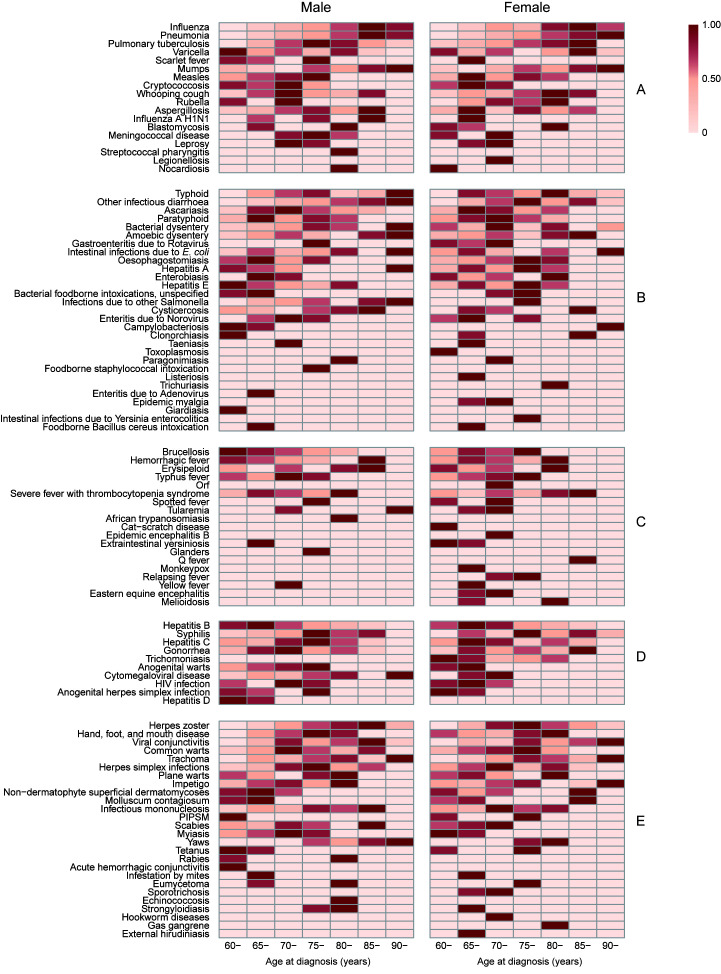
Incidence density of 102 infectious diseases, stratified by sex and age group. (**A**) Respiratory infectious diseases. (**B**) Gastrointestinal infectious diseases. (**C**) Vector-borne infectious diseases. (**D**) Blood- and sex-transmitted infectious diseases. (**E**) Mucocutaneous infectious diseases. The incidence density of each infectious disease is standardized from 0 to 1 according to the percentile rank and is represented by heat maps (with color scale from 0 to 1, where 1 is the highest incidence and 0 is the lowest incidence). PIPSM – picornavirus infections presenting in the skin or mucous membranes.

Our results demonstrated that males had a significantly higher incidence of the five categories of infectious diseases than females, in almost all age groups (**Table 3** and **Figure 3**). For respiratory diseases, males had an approximately 3-times higher risk compared with females (IRRa = 2.94, 95% CI = 2.89, 3.00). The incidence density increased with age in both genders, except for the age group of over 90 years (**Figure 3****,** Panel A). The incidence of elderly aged 85-89 years was nearly ten times higher than those aged 60-64 years (IRRa = 9.85, 95% CI = 9.39,10.33). In addition, the difference in incidence between males and females peaked at the 85-89 age group. As shown in **Figure 4**, Panel A, the incidences of influenza, pneumonia, and mumps were highest among elderly aged >80 years, while scarlet fever, measles, and rubella were highest among those aged <80 years.

**Table 3:** Incidence and associated demographic factors of the five categories of infections in Shandong province, China, 2013-2017

Variables	Cases	Person-years	Incidence density (per 100 000 PY)	Univariate analysis IRRc (95% CI)	Multivariate analysis IRRa (95% CI)
**Respiratory**					
**Gender:**					
Female	7059	1 045 214.92	675.36	1.00 (ref.)	1.00 (ref.)
Male	10 817	890 586.54	1214.59	1.80 (1.63, 1.97)*	2.94 (2.89, 3.00)*
**Age at diagnosis (years):**					
60-	2567	543 289.96	472.49	1.00 (ref.)	1.00 (ref.)
65-	3454	546 633.94	631.87	1.34 (1.19, 1.51)*	1.38 (1.30, 1.46)*
70-	2599	349 446.93	743.75	1.57 (1.40, 1.77)*	1.63 (1.54, 1.73)*
75-	2136	252 080.65	847.35	1.79 (1.60, 2.01)*	1.86 (1.75, 1.96)*
80-	3746	155 467.34	2409.51	5.10 (4.62, 5.64)*	5.24 (4.99, 5.51)*
85-	2802	64 314.23	4356.73	9.22 (8.33, 10.07)*	9.85 (9.39, 10.33)*
90-	572	24 568.41	2328.19	4.93 (4.48, 5.42)*	5.26 (5.00, 5.53)*
**Residence:**					
Rural	5878	850 264.03	691.31	1.00 (ref.)	1.00 (ref.)
Urban	11998	1 085 537.44	1105.26	1.60 (1.45, 1.76)*	3.30 (3.23, 3.37)*
**Gastrointestinal**					
**Gender:**					
Female	1241	1 045 214.92	118.73	1.00 (ref.)	1.00 (ref.)
Male	1305	890 586.54	146.53	1.23 (0.97, 1.57)	1.50 (1.41, 1.60)*
**Age at diagnosis (years):**					
60-	389	543 289.96	71.60	1.00 (ref.)	1.00 (ref.)
65-	838	546 633.94	153.30	2.14 (1.63, 2.86)*	2.22 (1.93, 2.56)*
70-	547	349 446.93	156.53	2.19 (1.67, 2.92)*	2.31 (2.01, 2.66)*
75-	418	252 080.65	165.82	2.32 (1.77, 3.06)*	2.45 (2.13, 2.83)*
80-	228	155 467.34	146.65	2.05 (1.55, 2.72)*	2.18 (1.89, 2.51)*
85-	89	64 314.23	138.38	1.93 (1.46, 2.59)*	2.17 (1.89, 2.50)*
90-	37	24 568.41	150.60	2.10 (1.60, 2.80)*	2.70 (2.35, 3.10)*
**Residence:**					
Rural	1215	850 264.03	142.90	1.00 (ref.)	1.00 (ref.)
Urban	1331	1 085 537.44	122.61	0.86 (0.67, 1.09)	0.78 (0.73, 0.83)*
**Vector-borne**					
**Gender:**					
Female	72	1 045 214.92	6.89	1.00 (ref.)	1.00 (ref.)
Male	98	890 586.54	11.00	1.59 (0.63, 4.35)	1.98* (1.52, 2.60)
**Age at diagnosis (years):**					
60-	43	543 289.96	7.91	1.00 (ref.)	1.00 (ref.)
65-	49	546 633.94	8.96	1.13 (0.43, 3.03)	1.14 (0.71, 1.84)
70-	38	349 446.93	10.87	1.37 (0.55, 3.56)	1.33 (0.85, 2.12)
75-	19	252 080.65	7.54	0.95 (0.34, 2.64)	0.96 (0.58, 1.58)
80-	14	155 467.34	9.01	1.14 (0.44, 3.03)	1.18 (0.74, 1.89)
85-	6	64 314.23	9.33	1.18 (0.45, 3.13)	1.25 (0.79, 2.00)
90-	1	24 568.41	4.07	0.51 (0.14, 1.63)	0.59 (0.33, 1.03)
**Residence:**					
Rural	72	850 264.03	8.47	1.00 (ref.)	1.00 (ref.)
Urban	98	1 085 537.44	9.03	1.07 (0.41, 2.78)	1.40 (1.09, 1.82)*
**Blood- and sex-transmitted**					
**Gender:**					
Female	562	1 045 214.92	53.77	1.00 (ref.)	1.00 (ref.)
Male	815	890 586.54	91.51	1.70 (1.22, 2.39)*	1.70 (1.53, 1.88)*
**Age at diagnosis (years):**					
60-	384	543 289.96	70.68	1.00 (ref.)	1.00 (ref.)
65-	475	546 633.94	86.90	1.23 (0.90, 1.68)	1.24 (1.06, 1.45)*
70-	256	349 446.93	73.26	1.04 (0.75, 1.43)	1.05 (0.89, 1.24)
75-	161	252 080.65	63.87	0.90 (0.64, 1.27)	0.89 (0.75, 1.05)
80-	76	155 467.34	48.88	0.69 (0.48, 0.99)*	0.71 (0.59, 0.86)*
85-	22	64 314.23	34.21	0.48 (0.32, 0.72)*	0.47 (0.38, 0.58)*
90-	3	24 568.41	12.21	0.17 (0.09, 0.31)*	0.14 (0.10, 0.20)*
**Residence:**					
Rural	416	850 264.03	48.93	1.00 (ref.)	1.00 (ref.)
Urban	961	1 085 537.44	88.53	1.81 (1.28, 2.58)*	2.09 (1.88, 2.33)*
**Mucocutaneous**					
**Gender:**					
Female	2957	1 045 214.92	282.91	1.00 (ref.)	1.00 (ref.)
Male	2669	890 586.54	299.69	1.05 (0.90, 1.25)	1.12 (1.08, 1.17)*
**Age at diagnosis (years):**					
60-	955	543 289.96	175.78	1.00 (ref.)	1.00 (ref.)
65-	1473	546 633.94	269.47	1.53 (1.27, 1.86)*	1.57 (1.42, 1.73)*
70-	1262	349 446.93	361.14	2.05 (1.72, 2.46)*	2.08 (1.90, 2.28)*
75-	981	252 080.65	389.16	2.21 (1.86, 2.64)*	2.22 (2.03, 2.44)*
80-	620	155 467.34	398.80	2.27 (1.90, 2.72)*	2.33 (2.13, 2.55)*
85-	256	64 314.23	398.05	2.26 (1.90, 2.71)*	2.38 (2.18, 2.61)*
90-	79	24 568.41	321.55	1.83 (1.52, 2.20)*	1.75 (1.59, 1.92)*
**Residence:**					
Rural	1475	850 264.03	173.48	1.00 (ref.)	1.00 (ref.)
Urban	4151	1 085 537.44	382.39	2.20 (1.85, 2.64)*	2.18 (2.09, 2.28)*

IRRc – crude incidence rate ratio, IRRa – adjusted incidence rate ratio, PY – person-years, CI – confidence interval

*P≤0.05.

For gastrointestinal infections, the highest incidence was among males aged >90 years and females aged 65-69 years (**Figure 3**, Panel B). In males, a significant increase with age in incidence of other infectious diarrhoea was seen (**Figure 4**, Panel B). The incidence of vector-borne diseases was relatively low in both genders, and there was no significant difference between age groups (**Figure 3**, Panel C). Blood- and sex-transmitted diseases showed similar distributions in both genders, with the peak incidence among elderly aged 65-69 years (**Figure 3**, Panel D, **Table 3**). In detail, however, the age-specific incidence of syphilis, gonorrhea, anogenital warts, and AIDS was highest among those aged 70-79 years for males, and among those aged 60-69 years for females (**Figure 4**, Panel D). The age distribution of mucocutaneous diseases differed by genders (**Figure 3**, Panel E). For females, mucocutaneous diseases were most common in elderly aged 75-79 years, whereas in males, the infections predominated in 85-89 age group. As shown in **Figure 4**, Panel E, this sex-specific age distribution was observed in cases of herpes zoster.

## DISCUSSION

In the present study, we used a multi-source big data platform to extensively investigate infectious diseases among the elderly. During the study period, 27 595 cases with 102 infectious diseases were identified from 550 432 elderly persons. With the objective of highlighting public health priorities, epidemiological characteristics and changes of these infectious diseases were summarised.

Overall, respiratory and mucocutaneous diseases were most common of the five categories. Progressive decline in immunity and additional chronic diseases are the physiological basis for the increased vulnerability to these infections [3,4]. Among all infections, influenza remained the top one infectious disease in incidence during the five years. Since influenza vaccination is not included in China’s National Immunization Program, the awareness and coverage rates of the vaccination are low, especially for elderly persons, which may be the main reason for the high incidence of influenza. During the 2011-2012 influenza season, the vaccination rate was 6.4% among China’s urban residents, and 4.3% among the elderly [16]. This coverage was far from its target of 75% for the elderly set by the World Health Assembly (WHA) in 2010 [17], and was much lower than that in other countries [18-20]. Previous studies showed that influenza-associated mortality increased with age [21]. Therefore, the elderly persons are a priority group for vaccination, who bear the greatest burden of influenza.

In addition, the incidence of pneumonia, cryptococcosis, whooping cough, and aspergillosis increased significantly during the study period, which can be attributed to the following reasons. First, diagnosis and reporting system of infectious diseases have been improved in these years, with fewer missing cases and missing reports. Second, increasing antimicrobial resistance, population growth and increasing human connectivity, changeable health-care seeking behaviour, as well as urbanization, may increase the threats of those infections [22]. Third, China has experienced severe and persistent air pollution in recent years, which is known to trigger and exacerbate various respiratory diseases [23,24]. In particular, antibodies to pertussis vaccination naturally disappear after seven to ten years, which may indicate the risk of resurgence of the disease and thus increase the incidence of pertussis in the elderly persons [25].

Herpes zoster, the most common mucocutaneous diseases among the elderly, showed a significantly increasing trend, which is consistent with previous studies domestic and abroad [8,26,27]. The reason of increased incidence of herpes zoster has not been concluded. Potential explanations for the increase in the incidence of herpes zoster might include ageing of the population, reduced immune function, previous VZV exposure, depression, and other diseases [27-29].

An alarming increase in the incidence of syphilis, mainly transmitted through sexual activity, was observed in the elderly individuals. Our results were in agreement with the passive surveillance reported by China Information System for Disease Control and Prevention (CISDCP) [30]. Similarly, serological studies of syphilis in other regions of China reported high prevalence of syphilis among the elderly [31]. The possible reasons may be due to the longer life expectancy, more common unsafe sex, infrequent use of condoms, and more medical screening. Additionally, more elder persons may be infected with syphilis by blood transmission, when they were operated in some informal healthcare providers for reducing expenses.

Therefore, targeted strategies and measures should be designed to prevent and mitigate the leading diseases, such as influenza and herpes zoster; as well as the diseases with increasing trends, including pneumonia, cryptococcosis, whooping cough, aspergillus, and syphilis. These measures include strengthening the screening for infectious diseases and vaccination among the elderly persons, reducing or exempting of treatment costs for infections, and implementing population-based public health interventions.

In the present study, the rate of all categories of infectious diseases in rural areas are very low in 2013-2014, which may be related to the incomplete health-care systems in rural areas. In addition, many rural residents would not go to the public hospitals because of the complex reimbursement procedure, so their medical records could not be found in our platform, resulting in a very low incidence in rural areas from 2013 to 2014. During the study period, an increase in the incidence of respiratory, gastrointestinal, blood- and sex-transmitted, and mucocutaneous diseases was identified in rural areas, which may be caused by the improvement of medical insurance in rural areas, the changes in the health-care behaviour of rural residents, and the aggravation of rural ageing. China has a huge agricultural population and there has been a large-scale migration of younger rural workers towards the cities in the process of urbanization. This has separated many adult children from their ageing parents (called empty-nesters), posing an increasingly severe ageing degree in rural areas, even more than in urban areas. As shown in previous studies, the loneliness may impact the physical and mental health of empty-nesters [32,33]; moreover, the elderly in rural areas may exhibit a relatively low socioeconomic status, characterised by inadequate dietary intake, poor hygiene and sanitation, poor access to health services and lack of education [34,35]. These factors may increase the incidence of infectious diseases in rural elderly.

Furthermore, incidence of infectious diseases has varied substantially between cities and regions. The possible reasons influencing the distribution may include the differences between culture, eating habits, and meteorological and environmental factors. In addition, public health programs, medical care infrastructures, and testing and reporting systems may also result in the variations in incidence among cities and regions. Therefore, it is necessary to enhance the fairness and accessibility of health services between different regions. Specific prevention and control measures should be introduced according to local conditions of infectious diseases.

Remarkably, age and gender have considerable influenced on respiratory infectious diseases. Ageing of the immune system was characterized by a decline in both adaptive and innate immune responses. In the present study, the elderly exhibited an increased risk of respiratory diseases with increasing age, which was in parallel with the previous studies. A systematic review identified age as a definite risk factor for community-acquired pneumonia (CAP) [36]. Another study confirmed that age and gender had a strong effect on overall incidence of CAP and pneumonia caused by various microbial pathogens [37]. Our results showed that incidence of respiratory infections was higher in elderly males than in females. The possible reasons are as follows. First, due to the differences of sex hormones and chromosomes, females tend to have a more robust and responsive immune responses than males [38,39]. Second, males are more susceptible to acute inflammation caused by infections than females [40]. Third, males are more adversely exposed to smoking and drinking habits, which may further impair their immune systems. Additionally, elderly males are more likely to be engaged in communal activities after retirement, increasing their exposure risk of respiratory infections.

This study also found the effects of age and sex on other categories of infectious diseases. For the elderly males over 80 years, prevention and control of respiratory and mucocutaneous infections must be top priorities in Shandong province, while for the younger elderly aged 60-79 years, the intervention of sexually transmitted diseases should be strengthened in both genders. By 2050, China’s elderly population aged over 65 years were estimated to hit 400 million (accounting for 26.9%), with 150 million over 80 years [41]. Our results showed that it is necessary to strengthen the health surveillance among the elderly persons, and provided an age- and gender-specific evidence for the infectious diseases control.

Several limitations of this study should be noted. First, the observation time is relatively short. Given some policies and measures will not take effect immediately, further studies with a longer observation time will be needed to confirm the findings of incidence trend. Second, the increased trend in incidence is due to a variety of factors and we could not conclude that these increases are attributed to the diseases itself. Moreover, only 550 432 elderly persons aged over 60 years in Shandong province were involved in the present study, thus limiting the generalisability of our findings to the elderly living in other regions of China.

## CONCLUSIONS

The challenges posed by infectious diseases have been continuously increasing among the elderly in Shandong province, China. Special attention should be paid to infectious diseases with high incidence and significant growth, such as influenza, herpes zoster, pneumonia, and syphilis. Fairness and accessibility to health care in rural areas should be improved. Top priorities for males over 80 years were respiratory and mucocutaneous infections, while for elderly aged 60-79 years was the sexually transmitted diseases. Therefore, targeted strategies and measures should be implemented in different regions and subgroups of this population.

## Additional material


Online Supplementary Document
