# Supplementary material for: Infectious diseases among elderly persons: Results from a population-based observational study in Shandong province, China, 2013-2017
Source: J Glob Health. 2021 Dec 25;11:08010. doi: 10.7189/jogh.11.08010 (PMC8710039; doi:10.7189/jogh.11.08010)
Supplement: Online Supplementary Document [file jogh-11-08010-s001.pdf]

Figure S1 Sampling procedures and integration of Shandong Multi-Center Healthcare Big Data Platform.

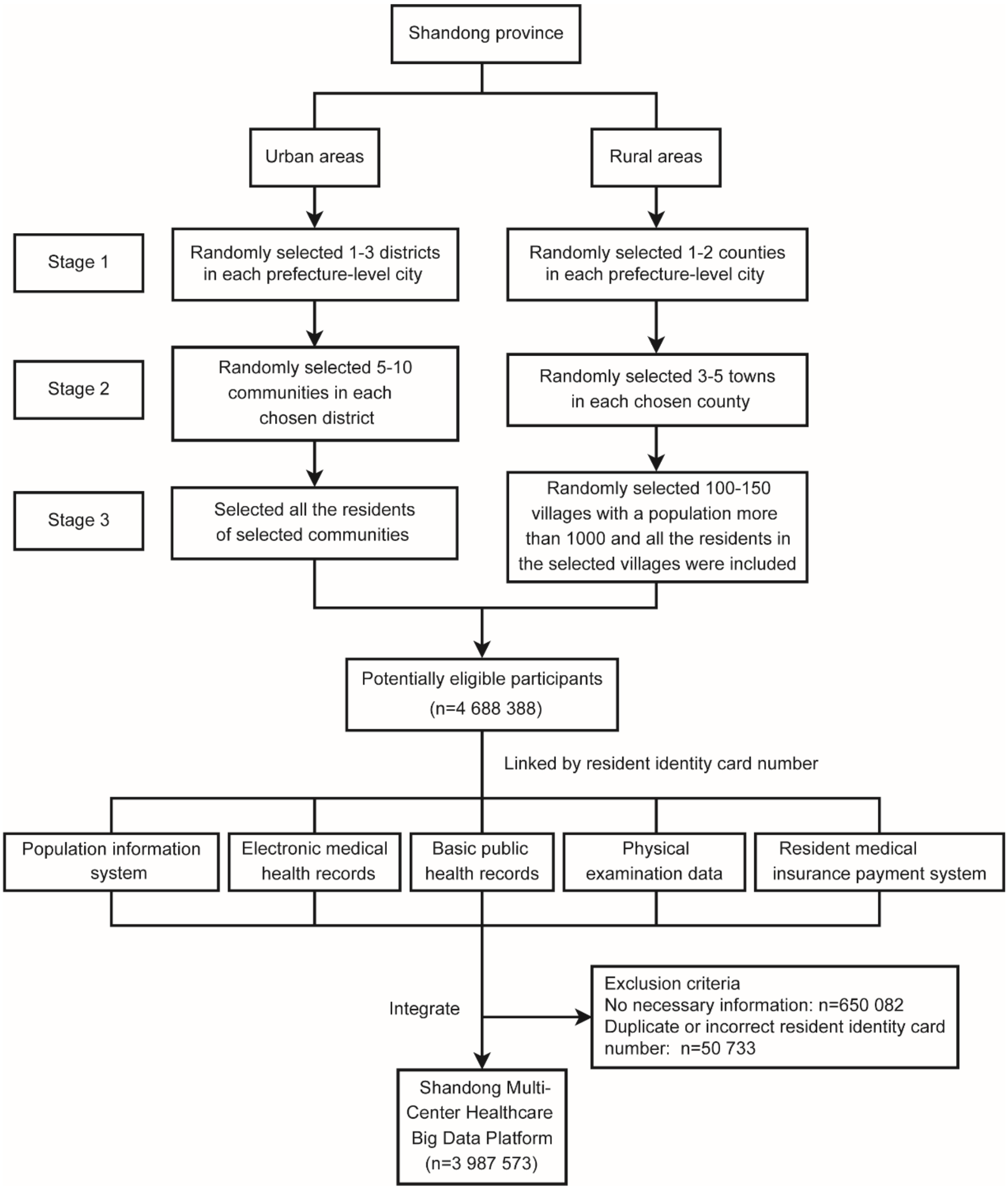

Table S1 Basic information of registered participants in "Shandong Multi-Center Healthcare Big Data Platform", January 2013 to June 2017

	Participants (n=3 987 573)
**Sex distribution of population, n (%)**	
Male	1 906 485 (47.81)
Female	2 081 088 (52.19)
**Residence**	
Urban	2 284 975 (57.30)
Rural	1 702 598 (42.70)
**Enrolment time (year), n (%)**	
2013	3 017 631 (75.67)
2014	338 850 (8.50)
2015	332 129 (8.33)
2016	214 279 (5.37)
2017	84 684 (2.12)
**Case number of infectious diseases (incidence density, per 100 000 PY)**	
Notifiable infectious diseases	73 801 (482.47)
Non-notifiable infectious diseases	32 488 (212.39)
**Case number of five categories of infectious diseases (incidence density, per 100 000 PY)**	
Respiratory	61 951 (405.00)
Gastrointestinal	9862 (64.47)
Vector-borne	1128 (7.37)
Blood- and sex-transmitted	10 170 (66.48)
Mucocutaneous	23 178 (151.52)
**Annual case number of infectious diseases (incidence density, per 100 000 PY)**	
2013	10 313 (364.81)
2014	12 813 (397.04)
2015	27 181 (771.20)
2016	35 167 (931.37)
2017	20 815 (1071.80)

PY – person-years

Table S2: Annual incidence and trends of 102 infectious diseases among the elderly in the population-based observational study of Shandong province, China, 2013-2017

Disease	Incidence density (per 100000 PY)					APC (%)
	2013	2014	2015	2016	2017	
**Respiratory diseases**	679.95	638.46	1001.44	1101.95	1156.70	17.45*
Influenza	537.39	558.96	822.54	838.70	828.51	13.56
Pneumonia	25.39	41.89	118.19	188.93	235.79	81.55*
Pulmonary tuberculosis	48.18	32.82	49.93	54.30	66.02	12.00
Varicella	63.83	0.25	1.08	1.18	0.38	-58.09
Scarlet fever	0	0.25	0.22	0.20	0.77	-
Mumps	0.65	0.25	1.73	0.79	1.53	-
Measles	2.28	1.01	2.38	0.79	0	-
Cryptococcosis	0	0.25	0.43	4.72	7.28	401.22*
Whooping cough	0.65	0.76	1.30	3.54	5.75	80.37*
Rubella	0.33	0.25	0	0.79	0.77	-
Aspergillosis	0.33	1.77	3.02	5.31	5.37	94.99*
Influenza A H1N1	0	0	0	0.59	0.77	-
Blastomycosis	0.33	0	0.22	0.79	0	-
Meningococcal disease	0	0	0.22	0.98	2.30	-
Leprosy	0.33	0	0	0.20	1.15	-
Streptococcal pharyngitis	0.33	0	0	0	0	-
Legionellosis	0	0	0	0.20	0	-
Nocardiosis	0	0	0.22	0	0.38	-
**Gastrointestinal diseases**	142.57	33.31	88.37	160.62	287.54	34.67
Typhoid	106.11	16.40	51.85	112.46	235.79	42.22
Other infectious diarrhoea	1.63	3.28	8.21	11.80	10.73	65.69*
Ascariasis	2.93	1.01	8.21	6.49	6.90	42.95
Paratyphoid	0.98	0	0.43	9.24	9.58	212.32
Bacterial dysentery	24.74	6.56	3.24	3.34	4.98	-32.17
Amoebic dysentery	0.98	1.01	4.75	5.50	4.60	61.40
Gastroenteritis due to Rotavirus	0	0	0	0.20	1.15	-
Intestinal infections due to *E. coli*	0	0	3.89	3.34	2.30	430.54
Oesophagostomiasis	0	0	0.22	2.16	4.22	-
Hepatitis A	1.30	0.25	1.51	0.98	0.77	-
Enterobiasis	0	0	0.65	0.79	0.38	-
Hepatitis E	1.95	2.78	2.16	0.79	0.77	-26.78
Bacterial foodborne intoxications, unspecified	0	0.50	0.43	0.59	0	-
Infections due to other Salmonella	0	0.25	0.22	0.39	1.53	-
Cysticercosis	0	0.50	0.22	0.98	2.30	-
Enteritis due to Norovirus	0.98	0.25	1.08	0.20	0	-

Campylobacteriosis	0.33	0	0	0.20	0.38	-
Clonorchiasis	0.33	0	0	0.20	0.38	-
Taeniasis	0	0	0	0.20	0.77	-
Toxoplasmosis	0	0	0.22	0	0	-
Paragonimiasis	0	0	0	0.39	0	-
Foodborne staphylococcal intoxication	0	0.25	0	0	0	-
Listeriosis	0	0	0	0.20	0	-
Trichuriasis	0.33	0	0	0	0	-
Enteritis due to Adenovirus	0	0	0.22	0	0	-
Epidemic myalgia	0	0	0.22	0.20	0	-
Giardiasis	0	0	0.22	0	0	-
Intestinal infections due to Yersinia enterocolitica	0	0	0.22	0	0	-
Foodborne Bacillus cereus intoxication	0	0.25	0.22	0	0	-
**Vector-borne diseases**	6.18	5.30	11.45	10.03	9.97	17.26
Brucellosis	0.33	2.78	2.38	3.54	5.75	81.44
Hemorrhagic fever	2.93	0.76	2.16	1.97	1.15	-8.77
Erysipeloid	0.33	0	1.51	1.18	0	-
Typhus fever	0.65	0.76	0.65	0.98	0.77	-
Orf	0	0	0.22	0	0	-
Severe fever with thrombocytopenia syndrome	0.65	0.25	2.59	1.18	0.38	-
Spotted fever	0	0	0.22	0.39	0.38	-
Tularemia	0	0	0.43	0.20	0.77	-
African trypanosomiasis	0	0.25	0	0	0	-
Cat-scratch disease	0	0	0.22	0	0	-
Epidemic encephalitis B	0.65	0	0	0	0	-
Extraintestinal yersiniosis	0.33	0.25	0	0.20	0	-
Glanders	0	0	0.22	0	0	-
Q fever	0	0	0.22	0	0	-
Monkeypox	0	0	0	0	0.38	-
Relapsing fever	0.33	0.25	0	0	0	-
Yellow fever	0	0	0	0.39	0	-
Eastern equine encephalitis	0	0	0.22	0	0.38	-
Melioidosis	0	0	0.43	0	0	-
**Blood- and sex-transmitted diseases**	65.75	52.49	75.41	80.61	79.75	8.49
Hepatitis B	49.16	41.65	56.21	49.98	49.91	2.15
Syphilis	2.28	4.54	4.32	13.37	18.02	68.45*
Hepatitis C	9.44	5.05	6.27	6.49	2.30	-22.69

Gonorrhea	2.28	0.25	5.19	5.90	4.98	60.38
Trichomoniasis	0	0	1.08	0.98	1.92	-
Anogenital warts	0.65	0.25	0.22	1.97	0.38	-
Cytomegaloviral disease	0	0.25	1.30	0.59	0.38	-
HIV infection	0.98	0.50	0.22	0.79	1.53	-
Anogenital herpes simplex infection	0.65	0	0.43	0.39	0.38	-
Hepatitis D	0.33	0	0.22	0.20	0	-
**Mucocutaneous diseases**	211.90	193.05	292.11	360.37	392.98	20.44*
Herpes zoster	122.06	136.02	222.76	277.21	295.60	28.16*
Hand, foot, and mouth disease	0.98	1.01	0.22	1.97	0.38	-
Viral conjunctivitis	26.04	20.95	31.98	36.76	43.32	17.12
Common warts	1.63	3.03	4.11	8.06	13.04	67.15*
Trachoma	47.52	19.94	19.01	15.33	17.64	-20.11
Herpes simplex infections	5.21	6.81	5.62	7.47	9.97	14.92
Plane warts	0	0	0.65	1.38	1.53	-
Impetigo	3.25	1.26	2.38	4.92	3.07	13.29
Non-dermatophyte superficial dermatomycoses	1.30	0.76	0.86	1.38	1.15	-
Molluscum contagiosum	0.65	0.25	0	0.79	1.92	-
Infectious mononucleosis	1.95	1.51	1.08	1.38	0	-
Picornavirus infections presenting in the skin or mucous membranes	0.33	0	0.22	0.20	0	-
Scabies	0	1.01	1.08	0.59	1.53	-
Myiasis	0	0	0.43	0.79	1.15	-
Yaws	0	0	0.43	0.20	1.53	-
Tetanus	0.65	0	0.22	0.39	0	-
Rabies	0	0	0.43	0	0	-
Acute hemorrhagic conjunctivitis	0.33	0	0	0	0	-
Infestation by mites	0	0	0	0.20	0.38	-
Eumycetoma	0	0	0.22	0.59	0.38	-
Sporotrichosis	0	0	0	0.20	0.38	-
Echinococcosis	0	0	0.22	0	0	-
Strongyloidiasis	0	0.25	0	0.39	0	-
Hookworm diseases	0	0	0.22	0	0	-
Gas gangrene	0	0	0	0.20	0	-
External hirudiniasis	0	0.25	0	0	0	-

APC – annual percentage change, PY – person-years

Note: Unavailable APC because of too few cases of the disease; * represent the trend test is statistically significant.

Table S3: The Top 20 infectious disease among the elderly in the population-based observational study of Shandong province, China, 2013-2017

Rank	2013		2014		2015		2016		2017	
	Disease	Incidence density*	Disease	Incidence density	Disease	Incidence density	Disease	Incidence density	Disease	Incidence density
1	Influenza	537.39	Influenza	558.96	Influenza	822.54	Influenza	838.70	Influenza	828.51
2	Herpes zoster	122.06	Herpes zoster	136.02	Herpes zoster	222.76	Herpes zoster	277.21	Herpes zoster	295.60
3	Typhoid	106.11	Pneumonia	41.89	Pneumonia	118.19	Pneumonia	188.93	Pneumonia	235.79
4	Varicella	63.83	Hepatitis B	41.65	Hepatitis B	56.21	Typhoid	112.46	Typhoid	235.79
5	Hepatitis B	49.16	Pulmonary tuberculosis	32.82	Typhoid	51.85	Pulmonary tuberculosis	54.30	Pulmonary tuberculosis	66.02
6	Pulmonary tuberculosis	48.18	Viral conjunctivitis	20.95	Pulmonary tuberculosis	49.93	Hepatitis B	49.98	Hepatitis B	49.91
7	Trachoma	47.52	Trachoma	19.94	Viral conjunctivitis	31.98	Viral conjunctivitis	36.76	Viral conjunctivitis	43.32
8	Viral conjunctivitis	26.04	Typhoid	16.40	Trachoma	19.01	Trachoma	15.33	Syphilis	18.02
9	Pneumonia	25.39	Herpes simplex infections	6.81	Ascariasis	8.21	Syphilis	13.37	Trachoma	17.64
10	Bacterial dysentery	24.74	Bacterial dysentery	6.56	OID	8.21	OID	11.80	Common warts	13.04
11	Hepatitis C	9.44	Hepatitis C	5.05	Hepatitis C	6.27	Paratyphoid	9.24	OID	10.73
12	Herpes simplex infections	5.21	Syphilis	4.54	Herpes simplex infections	5.62	Common warts	8.06	Herpes simplex infections	9.97
13	Impetigo	3.25	OID[†]	3.28	Gonorrhea	5.19	Herpes simplex infections	7.47	Paratyphoid	9.58

14	Hemorrhagic fever	2.93	Common warts	3.03	Amoebic dysentery	4.75	Hepatitis C	6.49	Cryptococcosis	7.28
15	Ascariasis	2.93	Hepatitis E	2.78	Syphilis	4.32	Ascariasis	6.49	Ascariasis	6.90
16	Measles	2.28	Brucellosis	2.78	Common warts	4.11	Gonorrhea	5.90	Whooping cough	5.75
17	Gonorrhea	2.28	Aspergillosis	1.77	Intestinal Infections due to *E. coli*	3.89	Amoebic dysentery	5.50	Brucellosis	5.75
18	Syphilis	2.28	Infectious mononucleosis	1.51	Bacterial dysentery	3.24	Aspergillosis	5.31	Aspergillosis	5.37
19	Hepatitis E	1.95	Impetigo	1.26	Aspergillosis	3.02	Impetigo	4.92	Gonorrhea	4.98
20	Infectious mononucleosis	1.95	Measles	1.01	SFTS[‡]	2.59	Cryptococcosis	4.72	Bacterial dysentery	4.98

Incidence density*: per 100 000 person-years.

OID - other infectious diarrhoea (infectious diarrhoeal diseases other than cholera, bacterial and amoebic dysentery, typhoid and paratyphoid); SFTS - severe fever with thrombocytopenia syndrome.
